# Are Soccer and Futsal Affected by the Relative Age Effect? The Portuguese Football Association Case

**DOI:** 10.3389/fpsyg.2021.679476

**Published:** 2021-05-28

**Authors:** Pedro Figueiredo, André Seabra, Marta Brito, Marta Galvão, João Brito

**Affiliations:** ^1^Portugal Football School, Portuguese Football Federation (FPF), Oeiras, Portugal; ^2^Research Center in Sports Sciences, Health Sciences and Human Development (CIDESD), University Institute of Maia (ISMAI), Maia, Portugal; ^3^Research Centre in Physical Activity, Health and Leisure (CIAFEL), Faculty of Sport, University of Porto, Porto, Portugal

**Keywords:** birthdate, football, player selection, gender, youth development

## Abstract

A better understanding of the relative age effect (RAE) in youth will increase the awareness of the need for reducing the bias of (de)selection. Thus, we investigated the RAE in youth female and male soccer and futsal players in Portugal, using nationwide data. Birthdates of 5,306 female and 126,285 male soccer players, and 2,437 female and 23,988 male futsal players (U7–U19), registered in Portugal during the season 2019–2020, and Portuguese National teams (from U15 to AA soccer teams and from U17 to AA futsal teams) were analyzed. Data were categorized into age groups and certification levels [no certification, basic football training center, football school, and training institution] of the respective clubs/academies. Birthdates were stratified from the start of the selection year using quartiles (Q) and semesters (S). Differences between the observed and expected birthdate distributions were analyzed using chi-square statistics, and RAEs were calculated using odds ratios (OR). In both soccer and futsal, female players, in the age category U9, RAEs were found (Q1 vs. Q4, OR: 1.49 and 1.84, respectively). In male soccer, differences in the birthdate distribution were observed in all age categories (U7–U19) with significant OR between all comparisons (Q and S). In contrast, an over-representation of young male futsal players (Q1 vs. Q4) was observed only in the age categories U7 and U9 (OR: 1.54 and 1.34, respectively). The stratification by certification level showed a significant RAE for all certification levels in male soccer players. In contrast, in male futsal players, the RAE was significant only in clubs and academies with the highest level. For National teams, the RAE was more pronounced in male soccer, particularly in the U16 and U17 (OR: 9.84 and 12.36, respectively). Data showed a RAE in female and male youth soccer and futsal, particularly in male, younger age categories, and in clubs and academies having a higher certification level, which could be accompanied by a loss of valuable elite players during the youth phase of their careers. Thus, adjustments in the systems and structure of talent identification are recommended to prevent RAE-related discrimination in youth soccer and futsal.

## Introduction

Youth athletic development is complex; it is a highly individual process and is affected by the interdependent factors within a constantly changing environment, such as physical growth, biological maturation, and behavioral development (Bergeron et al., 2015). Consequently, given the association of these factors with age, age plays a key role in this ongoing process.

In most countries, youth athletes are grouped based on chronological age cohorts with fixed cutoff dates aligned with the selection year (for example, January 1 to December 31), or in a window of 2 years (used by several sports organizations, including the Fédération International de Football Association, FIFA). Although this procedure is used to establish age-appropriate training, equalize competitive levels and reduce differences between opponents, it does not account for potentially large maturity-related differences that are possible within an age cohort (Helsen et al., 2005). This can effectively influence sporting talent identification and lead to an increased dropout from sport (Delorme et al., 2011; Breitbach et al., 2014).

Generally, a birthdate closer to the beginning of the year (e.g., in the first 3 months) has been associated with a sporting advantage, resulting in an over-representation of athletes born in that period. This has been defined as the relative age effect (RAE; Barnsley et al., 1985; Wattie et al., 2008). There is a widespread scientific opinion that advanced physical characteristics (e.g., greater body size and muscle mass, and better physical fitness) are most likely accountable for this over-representation of players born in the first quarter of the year (Malina et al., 2004, 2007; Cobley et al., 2009). A wider appreciation of the athletic triangle (i.e., coach, parent, and athlete) and factors beyond the physical should also be considered with regards to the RAE (Hancock et al., 2013; Wattie et al., 2015), particularly as data has demonstrated that the RAE is evident within pre-pubertal age groups, where maturity-related factors should not be a contributing factor (Doncaster et al., 2020).

At this stage, the Portuguese Football Association (FPF) is currently implementing a certification program that considers different levels of certification for youth development clubs and academies. The ultimate aim is to improve players’ development quality (up to the age of 19) at the club level, considering both the training process and the entire club or academy’s internal processes. Actually, the certification program is considered a priority project for the FPF, since in Portugal, soccer and futsal are popular sports, and the number of registered participants has been continuously increasing over the last years, both on female and male participants (Portugal Football Observatory, 2021).

The RAE has been extensively explored in soccer, but most studies have predominantly focused on professional elite male players, and less is known in other contexts such as female players and futsal athletes (Cobley et al., 2009; Smith et al., 2018). Of note, futsal is the official five-a-side indoor version of soccer. Though, findings are expected to vary according to the sporting context and type, and several other factors, including age, competition level, gender, and player position, are recognized as potential moderators of the RAE (Cobley et al., 2009; Smith et al., 2018). Based on the available evidence, gender and competition level are the most notable RAE moderators, but with inconsistent effects on both genders (Cobley et al., 2009; Romann and Fuchslocher, 2011; Sedano et al., 2015; Smith et al., 2018). Research suggests that the RAE is increasingly prevalent as the level of competition standard improves and on male athletes.

Some studies have explored the RAE using nationwide data (e.g., Finnegan et al., 2017; Romann et al., 2020; Dugdale et al., 2021). However, no study so far has analyzed original data at the level of a National sports governing body structure on both genders and football codes (i.e., soccer and futsal), and including data from National teams.

The purpose of this study was to investigate the RAE in female and male soccer and futsal youth players, across a range of age categories (from U7 to U19), and certification levels of clubs and academies registered in the FPF. Also, the RAE was analyzed in several Portuguese National Teams (soccer and futsal, male and female) from youth to professional adult players.

## Materials and Methods

### Participants and Procedures

In this study, we analyzed the birthdates of 5,306 female and 126,285 male soccer players, and 2,437 female and 23,988 male futsal players, registered with the FPF during the season 2019/2020. Players were categorized into age groups and certification level of their respective clubs or academies (3,018), as defined and classified by the FPF.^1^

The certification process from the FPF evaluates clubs and academies that provide training to young participants in soccer and futsal (up to the age of 19). The evaluation process is based on the following factors: strategic planning and budget; organizational structure and good practices; recruitment; sports training; medical support; school, personal and social monitoring; human resources; facilities and logistics; and, productivity. For the current study, we considered four levels of certification: no certification, basic football training center (BFTC), football school, and training institution.

The female and male Portuguese soccer and futsal National teams’ rosters were analyzed dating back to the season 2016/2017. In the FPF, National teams start from U15 to the adult professional level (i.e., AA). Players that were in more than one National Team at least twice were considered in both National Teams. Birthdate data are also publicly available on the internet.^2^ Players’ birthdates were collected from the FPF official database with permission and approval for treatment and analysis from the Portugal Football School and the Data Protection Office from the FPF.

The cutoff date used for the selection year, for all ages, was January 1st, as this is the same for all soccer and futsal leagues in Portugal, as well as for the National Teams. Birthdates for all players were stratified using quartiles (Q) and semesters (S). Thus, quartiles were organized as follows: from January to March (Q1), April to June (Q2), July to September (Q3), and from October to December (Q4), while S1 and S2 included the months from January to June, and July to December, respectively. The expected birthdate distributions were obtained from Statistics Portugal.^3^ The gender-, age-, and sport-specific reference population (RP) distributions were calculated considering the birth years from the youngest to the oldest players.

### Statistical Analysis

The observed birthdate distributions of all players were calculated for each quarter and semester of the year and presented as absolute and relative frequencies, for each age group, gender, and certification level in both soccer and futsal. Chi-square goodness-of-fit tests were used to compare the observed and expected birthdate distributions across quartiles. As Chi-squared statistics cannot reveal the magnitude and direction of an existing relationship, we additionally calculated the odds ratios (OR) and 95% CI for the quartiles (Q1, Q2, and Q3) and semester (S1), with the youngest group as reference (i.e., Q4 and S2). We also applied the Benjamini and Hochberg (1995) procedure for multiple testing correction, and reported the false discovery rate (FDR) adjusted *p*-values. FDR-adjusted *p*-values lower than 0.05 were assumed to be statistically significant. We assumed the existence of a RAE if the 95% CI range did not include a value ≤1, and interpreted an OR 1.22 ≤ OR < 1.86 as small, 1.86 ≤ OR <3.00 as medium, and OR ≥ 3.00 as large (Olivier and Bell, 2013). All statistical analyses were conducted using R statistical software (version 4.0.2, R Foundation for Statistical Computing, Vienna, Austria).

## Results

For the youth soccer and futsal players (age categories from U7 to U19), Table 1 displays the frequency and percentage distributions of players’ birth quartiles for each gender.

**Table 1:** Birthdate distribution by quarter of the year for female and male youth soccer and futsal players registered with the Portuguese Football Association.

Age category	*n*	Birthdate distribution, *n* (%)				*χ*^2^	FDR-adjusted *p*
		Q1	Q2	Q3	Q4		
Female							
*RP (2001–2015)*	*733,119*	*176,204 (24.0)*	*178,562 (24.4)*	*193,580 (26.4)*	*184,773 (25.2)*		
Soccer							
U7	180	46 (25.6)	52 (28.9)	53 (29.4)	29 (16.1)	8.23	0.096
U9	501	152 (30.3)	140 (27.9)	102 (20.4)	107 (21.4)	20.81	**<0.001**
U11	811	209 (25.8)	209 (25.8)	200 (24.7)	193 (23.8)	3.25	0.512
U13	904	226 (25.0)	242 (26.8)	219 (24.2)	217 (24.0)	4.65	0.353
U15	1,096	266 (24.3)	269 (24.5)	305 (27.8)	256 (23.4)	2.36	0.644
U17	1,031	243 (23.6)	270 (26.2)	273 (26.5)	245 (23.8)	2.36	0.644
U19	783	165 (21.1)	201 (25.7)	208 (26.6)	209 (26.7)	4.11	0.406
Futsal							
U7	60	16 (26.7)	16 (26.7)	13 (21.7)	15 (25.0)	0.82	0.846
U9	184	58 (31.5)	45 (24.5)	48 (26.1)	33 (17.9)	8.16	**0.043**
U11	257	71 (27.6)	59 (23.0)	67 (26.1)	60 (23.3)	1.95	0.583
U13	324	86 (26.5)	65 (20.1)	92 (28.4)	81 (25.0)	3.79	0.285
U15	424	110 (25.9)	113 (26.7)	109 (25.7)	92 (21.7)	3.70	0.295
U17	576	143 (24.8)	143 (24.8)	150 (26.0)	140 (24.3)	0.42	0.937
U19	612	166 (27.1)	131 (21.4)	162 (26.5)	153 (25.0)	4.63	0.201
Male							
*RP (2001–2015)*	*776,006*	*186,232 (24.0)*	*189,874 (24.5)*	*204,611 (26.4)*	*195,289 (25.2)*		
Soccer							
U7	6,759	1,876 (27.8)	1,770 (26.2)	1,772 (26.2)	1,341 (19.8)	124.15	**<0.001**
U9	15,348	4,199 (27.4)	3,812 (24.8)	3,844 (25.0)	3,493 (22.8)	118.56	**<0.001**
U11	23,372	5,923 (25.3)	5,808 (24.9)	6,048 (25.9)	5,593 (23.9)	35.28	**<0.001**
U13	23,824	6,080 (25.5)	5,738 (24.1)	6,356 (26.7)	5,650 (23.7)	45.21	**<0.001**
U15	22,502	6,100 (27.1)	5,710 (25.4)	5,620 (25.0)	5,072 (22.5)	176.43	**<0.001**
U17	19,851	5,408 (27.2)	5,089 (25.6)	4,930 (24.8)	4,424 (22.3)	181.22	**<0.001**
U19	14,629	3,959 (27.1)	3,777 (25.8)	3,624 (24.8)	3,269 (22.3)	128.46	**<0.001**
Futsal							
U7	1,285	385 (30.0)	319 (24.8)	318 (24.7)	263 (20.5)	31.66	**<0.001**
U9	2,708	753 (27.8)	689 (25.4)	676 (25.0)	590 (21.8)	31.72	**<0.001**
U11	3,856	921 (23.9)	930 (24.1)	1,024 (26.6)	981 (25.4)	0.38	0.944
U13	4,412	1,084 (24.6)	1,102 (25.0)	1,154 (26.2)	1,072 (24.3)	2.46	0.664
U15	4,519	1,091 (24.1)	1,075 (23.8)	1,185 (26.2)	1,168 (25.8)	1.76	0.751
U17	4,067	965 (23.7)	1,026 (25.2)	1,075 (26.4)	1,001 (24.6)	1.58	0.770
U19	3,141	732 (23.3)	757 (24.1)	880 (28.0)	772 (24.6)	4.48	0.362

FDR, false discovery rate; RP, reference population. Values in bold are significant.

In female players, the RAE was only evident within the U9 age category, in which the birthdate distribution differed significantly from the Portuguese population’s distribution. The descriptive OR comparisons are presented in Table 2. Both U9 female age categories (soccer and futsal) revealed significant but small OR for comparing Q1 and Q4, and between semesters.

**Table 2:** Odds ratios (OR; 95% CI) according to players’ age category by gender for youth soccer and futsal players examining relative age effect (RAE).

Age category	Odds ratios (95% CI)			
	Q1 vs. Q4	Q2 vs. Q4	Q3 vs. Q4	S1 vs. S2
Female				
Soccer				
U7	**1.66 (1.05–2.65)**	**1.86 (1.18–2.92)**	**1.74 (1.11–2.74)**	1.27 (0.95–1.71)
U9	**1.49 (1.16–1.91)**	**1.35 (1.05–1.74)**	0.91 (0.69–1.19)	**1.49 (1.25–1.78)**
U11	1.14 (0.93–1.38)	1.12 (0.92–1.36)	0.99 (0.81–1.21)	1.13 (0.99–1.30)
U13	1.09 (0.91–1.32)	1.15 (0.96–1.39)	0.96 (0.80–1.16)	1.14 (1.00–1.30)
U15	1.09 (0.92–1.29)	1.09 (0.92–1.29)	1.14 (0.96–1.34)	1.02 (0.90–1.15)
U17	1.04 (0.87–1.24)	1.14 (0.96–1.36)	1.06 (0.89–1.26)	1.06 (0.93–1.19)
U19	0.83 (0.67–1.02)	1.00 (0.82–1.21)	0.95 (0.78–1.15)	0.94 (0.81–1.08)
Futsal				
U7	1.12 (0.55–2.26)	1.10 (0.55–2.23)	0.83 (0.39–1.74)	1.22 (0.73–2.02)
U9	**1.84 (1.20–2.83)**	1.41 (0.90–2.21)	1.39 (0.89–2.16)	**1.36 (1.01–1.81)**
U11	1.24 (0.88–1.75)	1.02 (0.71–1.46)	1.07 (0.75–1.51)	1.09 (0.85–1.39)
U13	1.11 (0.82–1.51)	0.83 (0.60–1.15)	1.08 (0.80–1.46)	0.93 (0.75–1.16)
U15	1.25 (0.95–1.65)	1.27 (0.97–1.67)	1.13 (0.86–1.49)	1.18 (0.98–1.43)
U17	1.07 (0.85–1.35)	1.06 (0.84–1.33)	1.02 (0.81–1.29)	1.05 (0.89–1.24)
U19	1.14 (0.91–1.42)	0.89 (0.70–1.12)	1.01 (0.81–1.26)	1.01 (0.86–1.18)
Male				
Soccer				
U7	**1.47 (1.37–1.57)**	**1.36 (1.26–1.46)**	**1.26 (1.17–1.35)**	**1.25 (1.19–1.31)**
U9	**1.26 (1.20–1.32)**	**1.12 (1.07–1.18)**	**1.05 (1.00–1.10)**	**1.16 (1.12–1.20)**
U11	**1.11 (1.07–1.15)**	**1.07 (1.03–1.11)**	**1.03 (1.00–1.07)**	**1.07 (1.04–1.10)**
U13	**1.13 (1.09–1.17)**	**1.04 (1.01–1.08)**	**1.07 (1.04–1.11)**	**1.05 (1.02–1.07)**
U15	**1.26 (1.21–1.31)**	**1.16 (1.11–1.20)**	**1.06 (1.02–1.10)**	**1.17 (1.14–1.21)**
U17	**1.28 (1.23–1.33)**	**1.18 (1.14–1.23)**	**1.06 (1.02–1.11)**	**1.19 (1.16–1.23)**
U19	**1.27 (1.21–1.33)**	**1.19 (1.13–1.25)**	**1.06 (1.01–1.11)**	**1.19 (1.15–1.23)**
Futsal				
U7	**1.54 (1.31–1.80)**	**1.25 (1.06–1.47)**	1.15 (0.98–1.36)	**1.29 (1.15–1.44)**
U9	**1.34 (1.20–1.49)**	1.20 (1.08–1.34)	1.09 (0.98–1.22)	**1.21 (1.12–1.31)**
U11	0.98 (0.90–1.08)	0.98 (0.89–1.07)	1.00 (0.91–1.09)	0.98 (0.92–1.05)
U13	1.06 (0.97–1.15)	1.06 (0.97–1.15)	1.03 (0.95–1.12)	1.04 (0.98–1.11)
U15	0.98 (0.90–1.06)	0.95 (0.87–1.03)	0.97 (0.89–1.05)	0.98 (0.92–1.04)
U17	1.01 (0.93–1.10)	1.05 (0.97–1.15)	1.02 (0.94–1.12)	1.02 (0.96–1.08)
U19	0.99 (0.90–1.10)	1.01 (0.91–1.12)	1.09 (0.99–1.20)	0.96 (0.89–1.03)

Values in bold are significant.

In male soccer, results display a different distribution from the Portuguese population’s distribution in all age categories (Table 1). More players were born in the first quarters (i.e., over-represented), as revealed by the significant OR for the comparison between Q1 vs. Q4. The OR for the remaining comparisons were also significant, but with smaller magnitudes. Finally, male futsal results showed a different distribution from the Portuguese population’s distribution only in the two youngest age categories (U7 and U9). Again, we observed an over-representation of young male futsal players (U7 and U9) born in the first quarters, supported by the significant OR (1.54 and 1.34, respectively; Table 2).

Table 3 shows the distribution by quarters of the players’ dates of birth according to the different certification levels of clubs and academies.

**Table 3:** Birthdate distribution for female and male youth soccer and futsal players in Portugal according to the different certification levels of clubs and academies, as classified by the Portuguese Football Association.

Certification level	*n*	Birthdate distribution, *n* (%)				*χ*^2^	FDR-adjusted *p*
		Q1	Q2	Q3	Q4		
Female							
*RP (2001–2015)*	*733,119*	*176,204 (24.0)*	*178,562 (24.4)*	*193,580 (26.4)*	*184,773 (25.2)*		
Soccer							
No certification	4,490	1,108 (24.7)	1,187 (26.4)	1,139 (25.4)	1,056 (23.5)	15.63	**0.004**
BFTC	291	76 (26.1)	70 (24.1)	77 (26.5)	68 (23.4)	0.93	0.888
Football school	300	72 (24.0)	66 (22.0)	89 (29.7)	73 (24.3)	1.98	0.715
Training institution	225	51 (22.7)	60 (26.7)	55 (24.4)	59 (26.2)	1.09	0.874
Futsal							
No certification	2,211	593 (26.8)	512 (23.2)	583 (26.4)	523 (23.7)	10.55	**0.014**
BFTC	41	12 (29.3)	11 (26.8)	11 (26.8)	7 (17.1)	1.65	0.766
Football school	0	---	---	---	---		
Training institution	185	45 (24.3)	49 (26.5)	47 (25.4)	44 (23.8)	0.57	0.932
Male							
*RP (2001–2015)*	*776,006*	*186,232 (24.0)*	*189,874 (24.5)*	*204,611 (26.4)*	*195,289 (25.2)*		
Soccer							
No certification	74,083	19,265 (26.0)	18,336 (24.8)	19,131 (25.8)	17,351 (23.4)	224.54	**<0.001**
BFTC	6,850	1,810 (26.4)	1,753 (25.6)	1,741 (25.4)	1,546 (22.6)	41.01	**<0.001**
Football school	10,871	2,812 (25.9)	2,746 (25.3)	2,774 (25.5)	2,539 (23.4)	35.73	**<0.001**
Training institution	34,481	9,658 (28.0)	8,869 (25.7)	8,548 (24.8)	7,406 (21.5)	472.08	**<0.001**
Futsal							
No certification	17,678	4,295 (24.3)	4,318 (24.4)	4,655 (26.3)	4,410 (24.9)	1.01	0.880
BFTC	1,858	489 (26.3)	447 (24.1)	472 (25.4)	450 (24.2)	5.61	0.246
Football school	1,417	346 (24.4)	335 (23.6)	408 (28.8)	328 (23.1)	5.96	0.224
Training institution	3,035	801 (26.4)	798 (26.3)	777 (25.6)	659 (21.7)	26.43	**<0.001**

BFTC, basic football training center; FDR, false discovery rate; RP, reference population. Values in bold are significant.

Results for the female players showed that for both soccer and futsal, only in clubs and academies with no certification, the birthdate distribution differed significantly from the distribution in the RP, but the effect was weak (Table 4).

**Table 4:** Odds ratios (95% CI) by gender and certification level (according to the certification of clubs and academies as classified by the Portuguese Football Association.) for youth soccer and futsal players examining RAE.

Certification level	Odds ratios (95% CI)			
	Q1 vs. Q4	Q2 vs. Q4	Q3 vs. Q4	S1 vs. S2
Female				
Soccer				
No certification	**1.10 (1.01–1.20)**	**1.16 (1.07–1.26)**	1.03 (0.95–1.12)	**1.12 (1.05–1.18)**
BFTC	1.17 (0.84–1.63)	1.07 (0.76–1.49)	1.08 (0.78–1.50)	1.07 (0.85–1.35)
Football school	1.03 (0.75–1.43)	0.94 (0.67–1.31)	1.16 (0.85–1.59)	0.91 (0.72–1.14)
Training institution	0.91 (0.62–1.32)	1.05 (0.73–1.51)	0.89 (0.62–1.28)	1.04 (0.80–1.35)
Futsal				
No certification	**1.19 (1.06–1.34)**	1.01 (0.90–1.14)	1.06 (0.95–1.20)	1.07 (0.98–1.16)
BFTC	1.80 (0.71–4.57)	1.63 (0.63–4.19)	1.50 (0.58–3.87)	1,36 (0.74–2.53)
Football school	---	---	---	---
Training institution	1.07 (0.71–1.63)	1.15 (0.77–1.73)	1.02 (0.68–1.54)	1.10 (0.83–1.47)
Male				
Soccer				
No certification	**1.16 (1.14–1.19)**	**1.09 (1.06–1.11)**	**1.05 (1.03–1.08)**	**1.10 (1.08–1.11)**
BFTC	**1.23 (1.15–1.31)**	**1.17 (1.09–1.25)**	**1.07 (1.00–1.15)**	**1.15 (1.10–1.21)**
Football school	**1.16 (1.10–1.23)**	**1.11 (1.05–1.17)**	**1.04 (1.00–1.10)**	**1.11 (1.07–1.16)**
Training institution	**1.37 (1.33–1.41)**	**1.23 (1.19–1.27)**	**1.10 (1.07–1.14)**	**1.23 (1.21–1.26)**
Futsal				
No certification	1.02 (0.98–1.07)	1.01 (0.97–1.05)	1.01 (0.97–1.05)	1.01 (0.98–1.04)
BFTC	**1.14 (1.00–1.30)**	1.02 (0.90–1.16)	1.00 (0.88–1.14)	1.08 (0.99–1.18)
Football school	1.11 (0.95–1.29)	1.05 (0.90–1.22)	**1.19 (1.03–1.37)**	0.98 (0.89–1.09)
Training institution	**1.27 (1.15–1.41)**	**1.25 (1.12–1.38)**	**1.13 (1.01–1.25)**	**1.18 (1.10–1.27)**

BFTC, basic football training center. Values in bold are significant.

There was a significant RAE for all certification levels in male soccer players, and the OR only slightly declined across comparisons. In male futsal players, the RAE was significant only in clubs and academies with training institution certification.

The birthdate distributions by quarters and OR for the National Teams are presented in Tables 5 and 6, respectively.

**Table 5:** Birthdate distribution for female and male soccer and futsal players in Portuguese National Teams.

National team		Birthdate distribution, *n* (%)				*χ*^2^	FDR-adjusted *p*
	*n*	Q1	Q2	Q3	Q4		
Female							
*RP (1979–2006)*	*1,589,225*	*386,041 (24.3)*	*400,367 (25.2)*	*410,601 (25.8)*	*392,216 (24.7)*		
Soccer							
U15	56	19 (33.9)	17 (30.4)	14 (25.0)	6 (10.7)	7.17	0.144
U16	91	23 (25.3)	30 (33.0)	21 (23.1)	17 (18.7)	3.81	0.430
U17	82	26 (31.7)	23 (28.0)	17 (20.7)	16 (19.5)	3.84	0.430
U19	81	25 (30.9)	19 (23.5)	20 (24.7)	17 (21.0)	2.03	0.567
AA	57	6 (10.5)	23 (40.4)	17 (29.8)	11 (19.3)	10.66	**0.034**
*RP (1984–2007)*	*1,345,949*	*326,583 (24.3)*	*336,699 (25.0)*	*348,914 (25.9)*	*333,753 (24.8)*		
Futsal							
U17	30	10 (33.3)	9 (30.0)	6 (20.0)	5 (16.7)	2.52	0.664
U18	14	7 (50.0)	3 (21.4)	1 (7.1)	3 (21.4)	5.86	0.227
U19	28	12 (42.9)	7 (25.0)	4 (14.3)	5 (17.9)	6.00	0.224
AA	36	10 (27.8)	11 (30.6)	7 (19.4)	8 (22.2)	1.30	0.830
Male							
*RP (1978–2006)*	*1,864,240*	*451,770 (24.2)*	*473,909 (25.4)*	*480,733 (25.8)*	*457,828 (24.6)*		
Soccer							
U15	79	44 (55.7)	20 (25.3)	9 (11.4)	6 (7.6)	47.88	**<0.001**
U16	122	68 (55.7)	33 (27.0)	14 (11.5)	7 (5.7)	77.38	**<0.001**
U17	114	61 (53.5)	37 (32.5)	11 (9.6)	5 (4.4)	72.94	**<0.001**
U18	131	58 (44.3)	42 (32.1)	19 (14.5)	12 (9.2)	43.10	**<0.001**
U19	147	63 (42.9)	44 (29.9)	25 (17.0)	15 (10.2)	38.94	**<0.001**
U20	120	46 (38.3)	34 (28.3)	23 (19.2)	17 (14.2)	17.56	**0.002**
U21	80	32 (40.0)	21 (26.3)	12 (15.0)	15 (18.8)	12.94	**0.013**
AA	61	13 (21.3)	14 (23.0)	14 (23.0)	20 (32.8)	2.23	0.683
*RP (1979–2005)*	*1,723,059*	*417,166 (24.2)*	*437,945 (25.4)*	*444,923 (25.8)*	*423,025 (24.6)*		
Futsal							
U17	70	19 (27.1)	29 (41.4)	9 (12.9)	13 (18.6)	12.89	**0.013**
U18	26	6 (23.1)	12 (46.2)	4 (15.4)	4 (15.4)	6.40	0.196
U19	53	14 (26.4)	25 (47.2)	7 (13.2)	7 (13.2)	16.02	**0.003**
U21	48	12 (25.0)	18 (37.5)	10 (20.8)	8 (16.7)	4.45	0.362
AA	34	10 (29.4)	9 (26.5)	8 (23.5)	7 (20.6)	0.68	0.920

FDR, false discovery rate; RP, reference population. Values in bold are significant.

**Table 6:** Odds ratios (95% CI) by gender and National Team, organized by the Portuguese Football Association, for female and male soccer and futsal players.

National team	Odds ratios (95% CI)			
	Q1 vs. Q4	Q2 vs. Q4	Q3 vs. Q4	S1 vs. S2
Female				
Soccer				
U15	**3.22 (1.28–8.06)**	**2.78 (1.09–7.04)**	2.23 (0.86–5.80)	**1.84 (1.06–3.17)**
U16	1.37 (0.73–2.57)	1.73 (0.95–3.13)	1.18 (0.62–2.24)	1.42 (0.94–2.16)
U17	1.65 (0.89–3.08)	1.41 (0.74–2.67)	1.01 (0.51–2.01)	1.52 (0.97–2.36)
U19	1.49 (0.81–2.77)	1.09 (0.57–2.11)	1.12 (0.59–2.15)	1.21 (0.78–1.88)
AA	0.55 (0.20–1.50)	2.05 (0.99–4.20)	1.48 (0.69–3.15)	1.06 (0.63–1.78)
Futsal				
U17	2.04 (0.70–5.98)	1.78 (0.60–5.32)	1.15 (0.35–3.76)	1.78 (0.85–3.74)
U18	2.38 (0.62–9.22)	0.99 (0.20–4.91)	0.32 (0.03–3.07)	2.57 (0.81–8.20)
U19	2.45 (0.86–6.96)	1.39 (0.44–4.37)	0.77 (0.21–2.85)	2.17 (0.98–4.80)
AA	1.28 (0.50–3.24)	1.36 (0.55–3.39)	0.84 (0.30–2.31)	1.44 (0.74–2.80)
Male				
Soccer				
U15	**7.43 (3.17–17.44)**	**3.22 (1.29–8.02)**	1.43 (0.51–4.01)	**4.33 (2.47–7.59)**
U16	**9.84 (4.52–21.43)**	**4.55 (2.01–10.30)**	1.90 (0.77–4.72)	**4.88 (3.05–7.80)**
U17	**12.36 (4.97–30.77)**	**7.15 (2.81–18.19)**	2.10 (0.73–6.03)	**6.21 (3.66–10.53)**
U18	**4.90 (2.63–9.12)**	**3.38 (1.78–6.42)**	1.51 (0.73–3.11)	**3.27 (2.19–4.89)**
U19	**4.26 (2.42–7.47)**	**2.83 (1.58–5.09)**	1.59 (0.84–3.01)	**2.71 (1.89–3.90)**
U20	**2.74 (1.57–4.78)**	**1.93 (1.08–3.46)**	1.29 (0.69–2.41)	**2.03 (1.39–2.96)**
U21	**2.16 (1.17–3.99)**	1.35 (0.70–2.62)	0.76 (0.36–1.63)	**1.99 (1.25–3.16)**
AA	0.66 (0.33–1.32)	0.68 (0.34–1.34)	0.67 (0.34–1.32)	0.81 (0.49–1.33)
Futsal				
U17	1.48 (0.73–3.00)	**2.15 (1.12–4.14)**	0.66 (0.28–1.54)	**2.21 (1.34–3.67)**
U18	1.52 (0.43–5.39)	2.90 (0.93–8.98)	0.95 (0.24–3.80)	2.28 (0.99–5.25)
U19	2.03 (0.82–5.02)	**3.45 (1.49–7.98)**	0.95 (0.33–2.71)	**2.83 (1.54–5.21)**
U21	1.52 (0.62–3.72)	2.17 (0.95–5.00)	1.19 (0.47–3.01)	1.69 (0.94–3.03)
AA	1.45 (0.55–3.81)	1.24 (0.46–3.33)	1.09 (0.39–3.00)	1.29 (0.65–2.53)

Values in bold are significant.

The *χ*^2^-statistics showed a significant difference between the relative age quarter distributions compared with the reference population only in the female AA soccer team, the male U19 futsal team, and in all male soccer teams (except the AA team). However, there was a stronger over-representation of young male soccer players born in the first quarters in the U16 and U17 teams, as OR progressively declined as the teams’ age category increased (Table 6).

## Discussion

This study investigated the RAE in soccer and futsal players register in Portugal across several age categories, according to gender and certification level of clubs and academies registered with the FPF. The analysis also included data from players selected for National teams. The major findings of this study showed that: (i) in male soccer players, there was a statistically significant RAE in all youth age groups (including the National teams from U15 to U21); (ii) in male futsal players, the RAE was observed only in the younger age groups (U7 and U9); (iii) the RAE was less prevalent among females; and (iv) although the RAE was found among male soccer players irrespectively of the certification levels, it was more prevalent in the clubs and academies classified in the highest level of certification.

The results presented here are consistent with those from prior studies showing that the RAE is prevalent and persistent in male players worldwide (Cobley et al., 2008; Jimenez and Pain, 2008; Mujika et al., 2009; Williams, 2010). Though, the RAE has also been found in female athletes, but results are still unclear regarding the real effect on soccer and futsal. In fact, while some studies have reported no significant RAE (Delorme et al., 2009; Romann and Fuchslocher, 2011; Júnior et al., 2018), the aggregated results reported in a recent meta-analysis supported a small RAE for soccer (OR = 1.31; Smith et al., 2018). Consistent with these findings, we only detected significant RAE in the female age category U9 (soccer and futsal). This over-representation of players born at the beginning of the year for one of the youngest age groups (U9) lends further support to the idea suggested in previous studies that initial enrolment bias facilitated by parents may explain the RAE at early ages (Hancock et al., 2013). Also, it is possible that the difference observed by gender on RAE can be related to the level of attraction of a sport for girls and boys and variations of competition levels (Baker et al., 2010). Götze and Hoppe (2021) suggest that other reason could be a less intense competition within a team to make the starting line-up.

In the current study, there was a predominance of the RAE in male soccer compared with the effect found in male futsal players. These divergent findings could be related to factors such as the sport’s popularity within Portugal or physiological and psychological conditions (Musch and Grondin, 2001; Cote et al., 2006; Mccarthy and Collins, 2014). In addition, sports regarded as popular are likely to increase competitiveness levels from an early age, which has been shown to exacerbate the RAE (Bezuglov et al., 2019). At the club level, Doncaster et al. (2020) found a RAE within a range of sports, but more prevalent in sports that may be regarded as popular, such as male soccer. It has been argued that if the process of talent identification is focused on the short-term success, it may contribute to this pattern. However, it should be acknowledged that the determinants and requirements for success in top-level soccer are non-linear and multifactorial (Skorski et al., 2016). Also, coaches’ role in the genesis of RAEs and their subsequent amplification has been highlighted (Hancock et al., 2013). As key social agents, coaches influence RAE through Pygmalion effects, i.e., wrongly influenced and based on physical maturity, coaches may develop higher expectations for relatively older children compared to peers (Hancock et al., 2013).

Our findings indicated that the RAE was not present in the male adult professional soccer team (National AA-Team), and the OR were higher between Q1 and Q4 in U16 and U17 than in younger and older age groups. These results were expected because in professional adult teams all players should be able to perform at a high level (i.e., comparable levels in physical performance), have small disparities in growth and maturation (Lovell et al., 2015), and high levels of exposure to training. All these factors should be considered in relation to the reduced prevalence of the RAE. Moreover, a longitudinal investigation into the RAE in an English professional soccer club showed that Q4 male soccer players were approximately four times more likely to achieve adult professional status than Q1 player’s, despite the reduced number of players within Q4 (Kelly et al., 2019). This reinforces the changes associated with the transition from youth to professional adult level, which has implications in the RAE, as reported by others (Brustio et al., 2018; Lupo et al., 2019; Dugdale et al., 2021).

In youth soccer players from Portuguese clubs and academies, the RAE was also observed across all age categories, but the highest OR was found between Q1 and Q4 in U7 soccer players. Similar to these results, prior studies have also reported that the extent of the RAE decreases with increasing age (Helsen et al., 2005; Lovell et al., 2015; Doncaster et al., 2020). There is evidence that the RAE decreases with age after adolescence (Cobley et al., 2009; Brustio et al., 2018). Actually, for the German national male youth soccer teams, it was reported that the RAE decreased with increasing age categories from U16 to the adult professional team (Skorski et al., 2016). Also, our finding highlights that the RAE is evident within pre-pubertal age groups, where maturity-related factors should not be a contributing factor, as also showed by Doncaster et al. (2020).

The lower prevalence of the RAE within futsal may be explained by the fact that futsal is less popular in Portugal than soccer, taking into account the broad differences in the number of registered players in both sports (according to 2019 data from registered male players, from the overall male Portugal population aged 5–19 years, 22% play soccer, while only 5% play futsal). Also, some players may have transitioned to futsal after a period in soccer where they did not excel, but they developed physical attributes, which may dilute the maturity disparities associated with chronological age differences (Lago-Fuentes et al., 2020). Also, as the official futsal laws of the game^4^ allow unlimited changes of players during the match, less mature players might have more chances to develop technical and tactical skills, offsetting their physical disadvantages when compared with more mature peers.

Finally, our findings indicated that in male soccer and futsal, the RAE extension increased as the certification level of clubs and academies improved. This was particularly highlighted in soccer. This might suggest that clubs and academies certified as a training institution also have the means to select more the players than the lower level certification clubs and academies, thus taking advantage of the potential beneficial effect of an over-representation of the chronologically older players. No comparisons with other studies are possible, as this is the first study reporting the RAE according to each club or academy classification level, which is attributed based on a specific certification process implemented by the FPF. Of note, when looking into the RAE on male Scottish youth soccer players, Dugdale et al. (2021) showed an influence of the playing level within male soccer academy structures.

In Portugal, clubs and academies are the primary talent development settings in soccer and futsal. Though, the decisions made about who is selected to be part of these structures at an early age will impact the subsequent long-term outputs from the sports development systems or programs implemented. Therefore, these findings are essential to better understand why specific individuals might be more likely to be selected into a soccer or futsal team. For example, in a nationwide analysis of Swiss child and youth football players, Romann et al. (2020) found a RAE at the grassroots level and that the use a selection system seems to increase RAEs created by coach’s selection. Also, Dugdale et al. (2021) on their analysis of playing levels and ages of male Scottish youth soccer players found a bias in selecting individuals born earlier in the selection year within academies, but not at amateur level.

The current study supports and extends upon the existing RAE literature including a comprehensive overview of birthdate distributions across multiple age groups in both female and male soccer and futsal players, alongside the National teams. This was a complete nationwide database, with large sample size, and the certification level attributed to youth clubs and academies was also examined as a factor potentially associated with the RAE. However, the absence of anthropometric and performance data, which could provide a better description of the RAE phenomenon, is a limitation of this study.

## Conclusion

Understanding the prevalence of RAE across several age categories in both female and male soccer and futsal players in a nationwide analysis will contribute to the discussion and implementation of national strategies for reducing this bias and other confounding factors of (de)selection. Also, it contributes toward a more systematic approach to talent identification and development in the plural contexts of the different certification levels of the clubs and academies, which are responsible for the players’ development. This study highlighted an observed RAE in male soccer, young male futsal players, and young female soccer and futsal players in Portugal. Interestingly, the RAE was observed in male soccer players for clubs and academies irrespectively of the certification level, although with a higher effect on the highest certification level. In male futsal players, the RAE was detected only in clubs and academies with training institution certification. For National teams, the RAE was prevalent in all soccer male teams from the U15 to U21. Despite the descriptive nature of the data, the results show possible questions for future research and highlight the need for an improved understanding of the factors influencing the RAE at a national level, beyond physical characteristics, using an integrated approach.

## Data Availability Statement

The data analyzed in this study is subject to the following licenses/restrictions: data was obtained from a third party. Requests to access the data analyzed in this study should be directed to Data Protection Office, Portuguese Football Association (dpo@fpf.pt).

## Ethics Statement

The studies involving human participants were reviewed and approved by the Portugal Football School and the Data Protection Office from the Portuguese Football Association. Written informed consent from the participants’ legal guardian/next of kin was not required to participate in this study in accordance with the national legislation and the institutional requirements.

## Author Contributions

PF, JB, and AS contributed to the conceptualization and design of the study. MG and MB performed the data collection. PF and MB performed the data analysis. PF wrote the original draft. All authors contributed to the article and approved the submitted version.

### Conflict of Interest

The authors declare that the research was conducted in the absence of any commercial or financial relationships that could be construed as a potential conflict of interest.

## Footnotes

^1^www.fpf.pt/Institucional/Documentação

^2^www.fpf.pt/Jogadores

^3^www.ine.pt

^4^https://www.fifa.com
